# Functional MRI to quantify perfusion changes of a renal allograft after embolization of an arteriovenous fistula

**DOI:** 10.1007/s40620-022-01539-y

**Published:** 2023-01-25

**Authors:** Cecilia Liang, David J. Heister, Martina Guthoff, Gerd Grözinger, Petros Martirosian, Ferdinand Seith

**Affiliations:** 1 Diagnostic and Interventional Radiology, University Department of Radiology, University Hospital of Tuebingen, Tuebingen, Germany; 2 Department of Nephrology, University Hospital of Tuebingen, Tuebingen, Germany; 3 Section on Experimental Radiology, Diagnostic and Interventional Radiology, University Department of Radiology, University Hospital of Tuebingen, Tuebingen, Germany

**Keywords:** Arterial spin labeling, Transplant kidney, Diffusion weighted imaging, Perfusion imaging, Arteriovenous fistula

## Abstract

Acute allograft injury was observed in a 37-year-old woman within a few weeks after kidney transplantation. Neither renal ultrasound nor computerized tomography (CT) and magnetic resonance (MR) angiography revealed any anomaly. An MR protocol was then performed including arterial spin labeling and intravoxel incoherent motion diffusion weighted imaging. Both arterial spin labeling and the perfusion fraction in the diffusion weighted imaging showed decreased perfusion compared to reference values. The patient subsequently underwent angiography, where an arteriovenous fistula in the upper calix of the transplant kidney was detected and immediate embolization was performed. A second functional MR, performed one week later, demonstrated a 40% increase in organ perfusion. We conclude that functional MR with arterial spin labeling and intravoxel incoherent motion have the potential to provide complementary information of clinical value to conventional imaging for monitoring renal allografts.

## Introduction

After solid organ transplantation, routine follow-up care is indispensable to identify early signs of allograft injury and to preserve long-term allograft function. Graft monitoring using plasma creatinine, proteinuria and ultrasonography are standard of care but sometimes might not be sensitive enough to detect complex pathophysiological processes, especially at early stages [1]. Renal biopsy is a gold standard of diagnostics and can provide further information about histopathological changes implying the risk of iatrogenic complications. In certain cases there is a need for sensitive quantification of perfusion changes in renal allografts, and multiparametric functional magnetic resonance imaging (MRI) could open up new possibilities in this field [2]. Without the administration of contrast media, MRI offers both morphological and functional imaging including techniques such as arterial spin labeling (ASL) or diffusion-weighted imaging (DWI). Unlike other imaging techniques, functional MRI provides the possibility to quantify measurements. Over the last decades, on-going technical developments in image quality and quantitative accuracy, as well as an increasing number of clinical studies have facilitated the application of functional MRI to native and transplant kidneys. Still, more clinical studies are required to prove its clinical value and enable the translation into clinical routine diagnostics for transplant kidneys.

## Case presentation

**Fig. 1:**
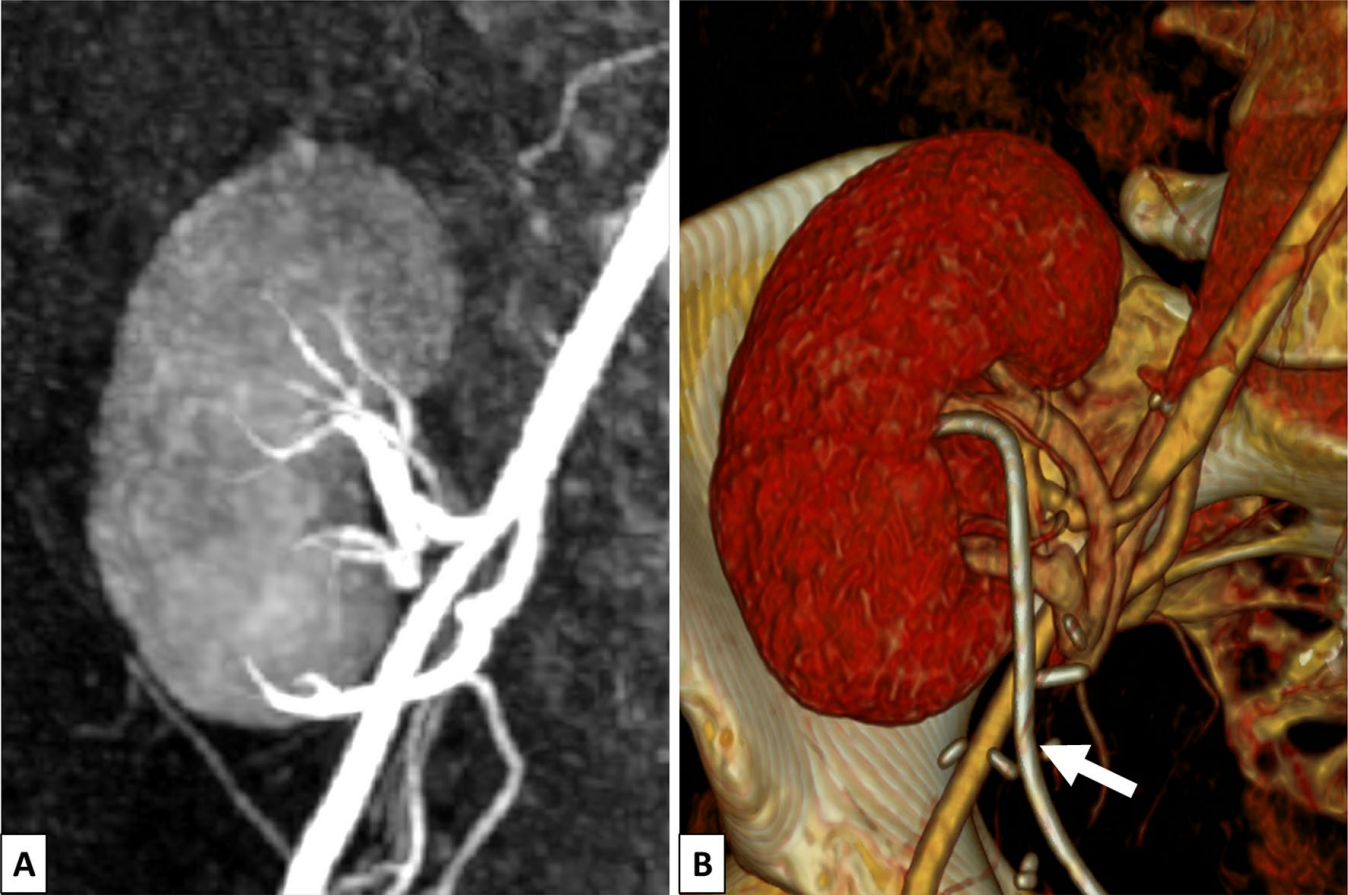
MR angiography (**A**) and CT angiography (**B**) with 3D reconstruction of the transplant kidney. The fistula could not be detected in either examination. At the time of the CT angiography, a double J-stent was implanted (white arrow)

A 37-year old woman showed deterioration in renal allograft function shortly after transplantation of a living-donor kidney. No anomalous changes were detected during regular Doppler ultrasound examinations. A renal biopsy performed less than 1 month after transplantation revealed no evidence of transplant rejection, but it did show severe acute tubular epithelial damage indicative of impaired perfusion. Alerted by the biopsy results, MR angiography was ordered followed by CT angiography to preclude any vascular complications (see Fig. 1). However, again no anomalies could be detected. A special MRI protocol was then performed to image functional changes including ASL and intravoxel incoherent motion (IVIM) DWI.

## Multiparametric functional MRI

A multiparametric functional MRI without contrast media was performed on a 3 T MRI scanner (MAGNETOM Prisma^fit^, Siemens Healthcare AG, Erlangen, Germany). The patient gave written informed consent for the examination and the publication of the results. Imaging protocol included perfusion imaging with ASL and DWI with IVIM. For ASL measurements, a flow-sensitive alternating inversion recovery technique was applied in combination with a true fast imaging with steady precession data acquisition strategy [3]. For IVIM measurements, reduced field of view diffusion-weighted echo planar imaging with multiple *b*-values was performed. Additionally, high resolution morphological T1 and T2 weighted images were included. Scan time took approximately 15 min. Images were acquired in free breathing. Region of interest-based image analysis was performed by manual segmentation of cortex and medulla. Renal blood flow (RBF) and perfusion fraction (PF) maps were calculated from ASL and IVIM data, respectively. Data were evaluated using MATLAB software (The MathWorks, Natick, MA).

**Fig. 2:**
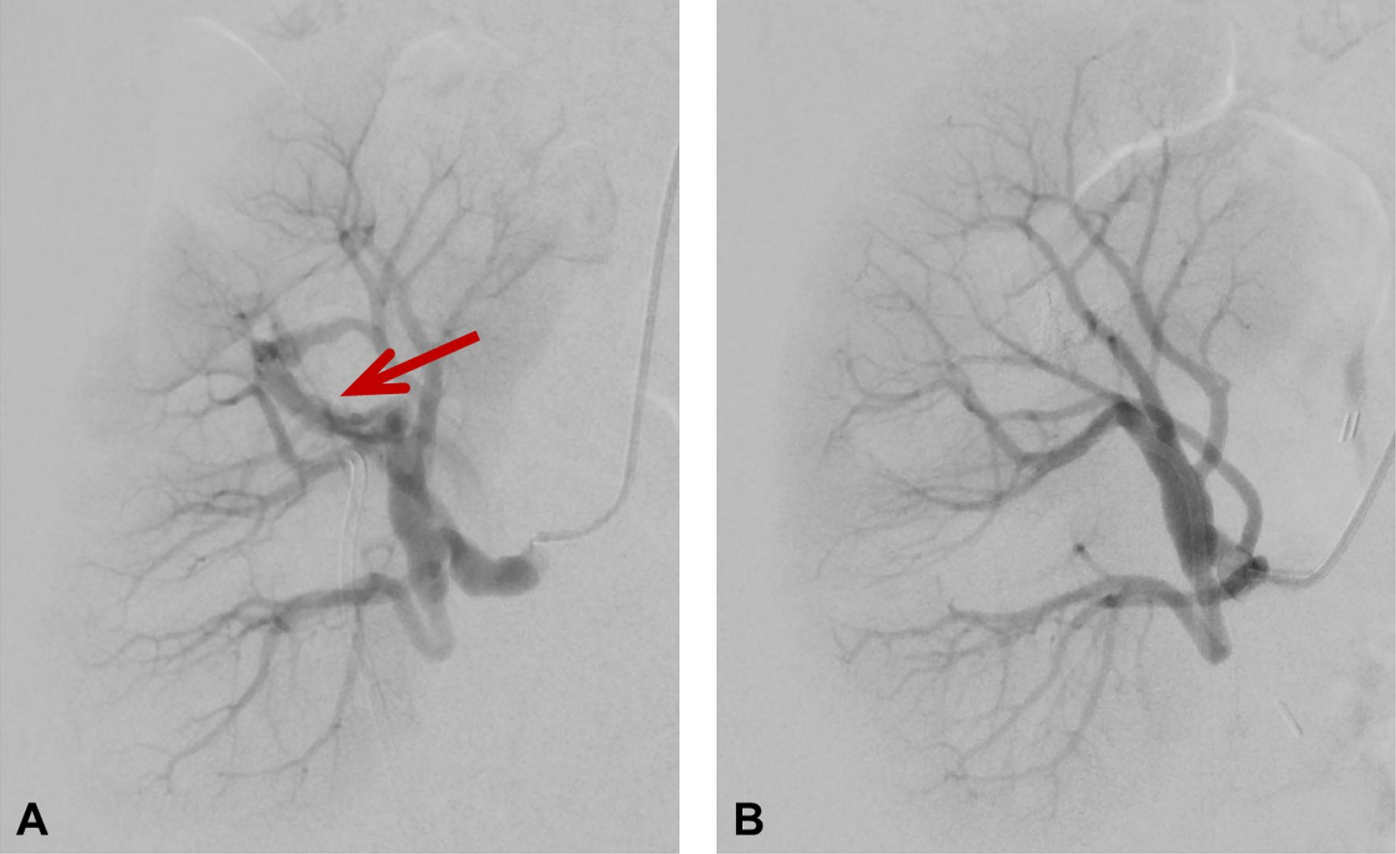
Digital subtraction angiography of the transplant kidney **A** before embolization with a red arrow pointing to the arteriovenous fistula in the upper calyx and **B** after embolization of the fistula. In **A** temporary vasospasm during angiography induced by the wire might have caused temporary vasospasm of the renal artery appearing as wall irregularities, but no manifest renal artery stenosis in this area was detected

**Fig. 3:**
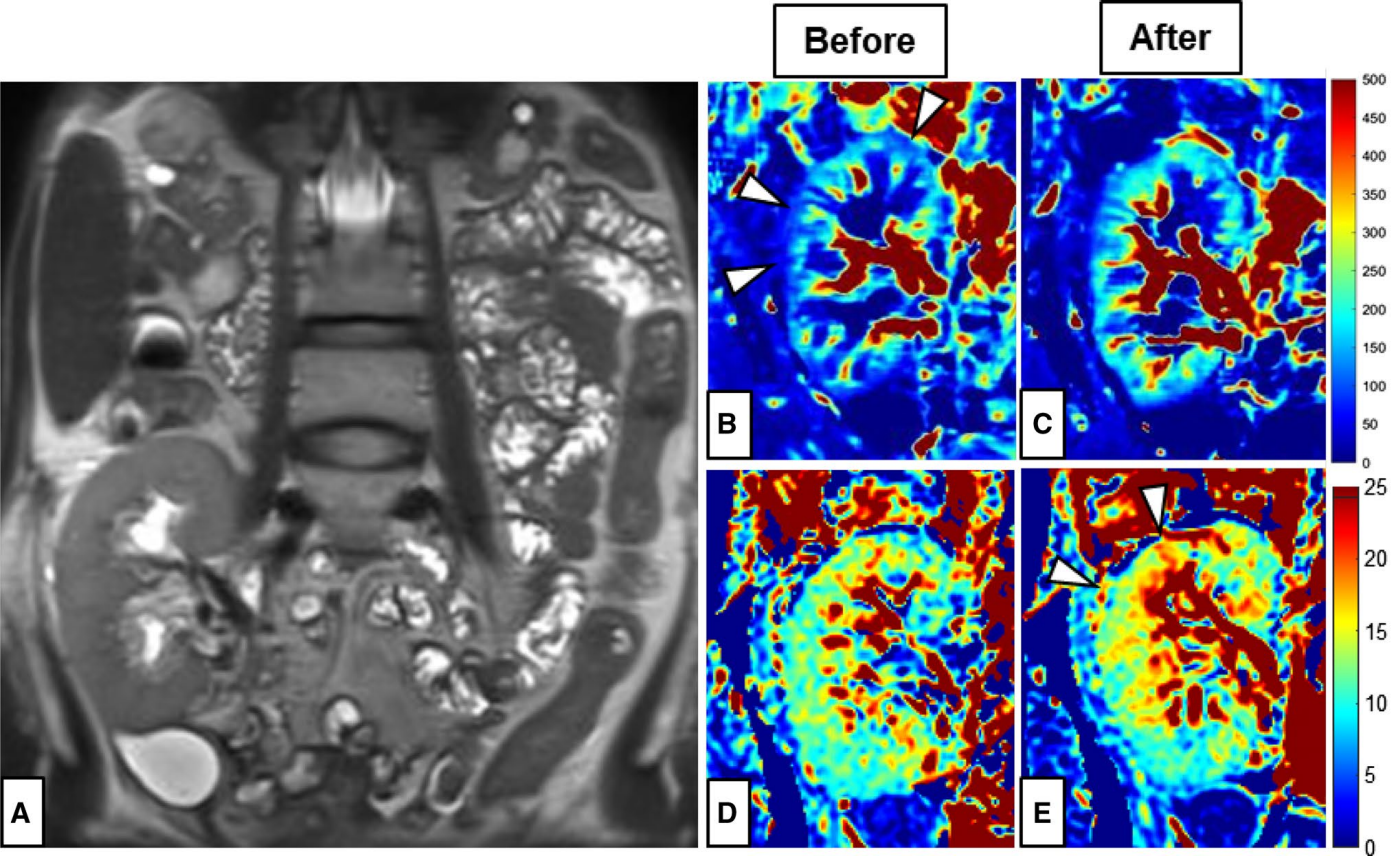
Functional MRI of the transplant kidney in the right iliac fossa: **A** Anatomical T2-weighted MR image depicting the renal artery (red arrow); RBF-maps in ml/min/100 g calculated from the ASL MRI before (**B**) and after (**C**) embolization; Perfusion fraction (PF) in % before (**D**) and after (**E**) embolization. Differences between measurements before and after embolization are highlighted by the white arrows pointing to the cortical perfusion deficit in the RBF map (**B**) and the improved cortical perfusion in the PF map in (**E**)

**Fig. 4:**
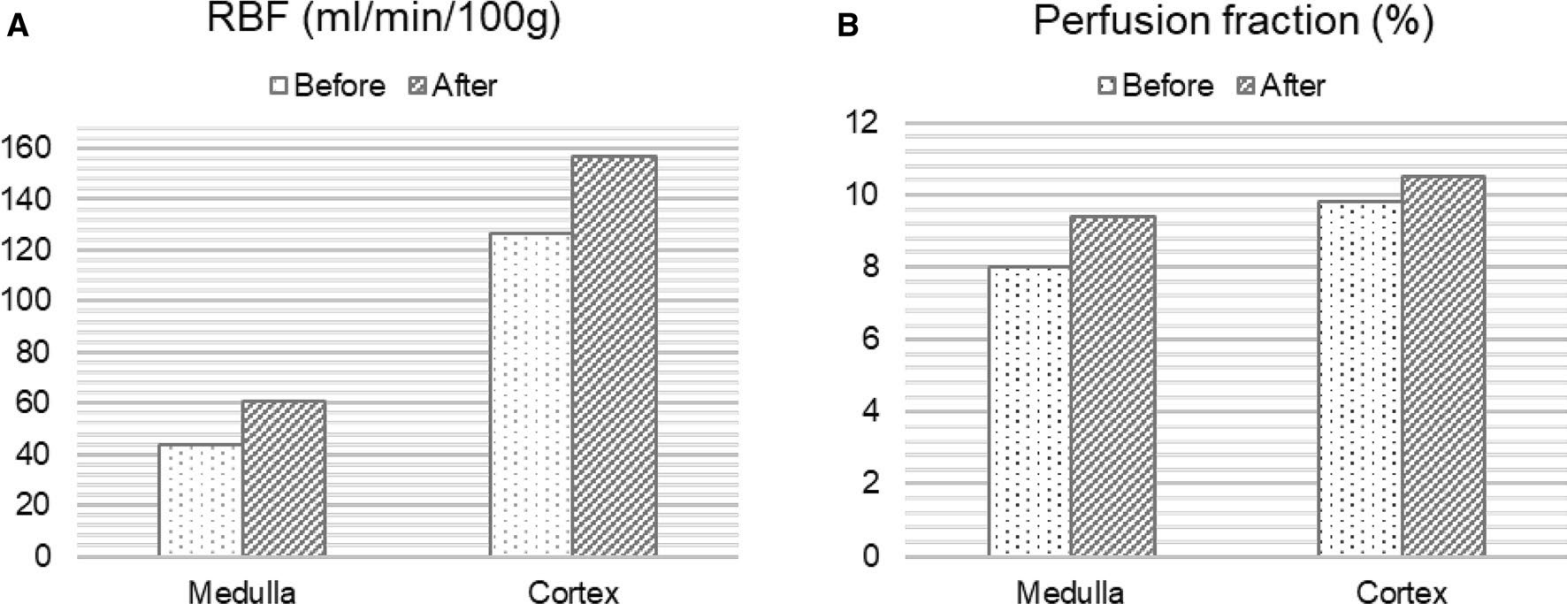
Calculation of the overall renal blood flow (RBF) in ml/min/100 g from the ASL MRI and the perfusion fraction in % from the IVIM MRI before and after embolization of the arteriovenous fistula

Perfusion measurement of the transplant kidney with ASL resulted in relatively low mean perfusion values for cortex with 127 ml/100 g/min and 44 ml/100 g/min for medulla compared to reference values between 200 and 400 ml/100 g/min for renal allografts with good function [4]. The PF acquired from the diffusion weighted imaging was also relatively low with approximately 8% in the cortex and 9.4% for medulla compared to reference values around 30% [5]. After interdisciplinary consultation, angiography was performed to visualize vascular supply of the renal allograft. Hereby, an arteriovenous fistula (AVF) was detected in the upper calyx (Fig. 2 and supplementary material). The hemodynamic relevance of the fistula set the indication for embolization, which was successfully performed immediately using a fluid polymer (Squid 18 (Squid^®^, Emboflu, Balt), an ethylene–vinyl alcohol copolymer-based liquid embolic agent) [6]. Five days after the intervention, a follow-up functional MRI was performed with the same parameters as the first MRI examination. This time, perfusion measurement with ASL showed a 23% increase in perfusion for the cortex and a 38% increase for the medulla. Likewise, the PF of IVIM was significantly elevated to 10.5% in the cortex and 9.8% in the medulla. The quantitative RBF images before and after the intervention are shown in Fig. 3. Figure 4 depicts the perfusion change in quantitative analysis of the RBF map and the PF.

## Discussion

Multiparametric functional MRI including ASL and IVIM approaches can noninvasively provide quantitative functional tissue information that goes beyond CT and MR angiography or Doppler ultrasound. In the present case, this additional information revealed reduced parenchymal perfusion of a kidney transplant with deteriorated function. After the embolization of an AVF, the parenchymal blood flow significantly improved as shown by functional MRI.

ASL is a non-invasive MRI technique for perfusion imaging without the need for contrast media. Instead, water protons in the arterial blood are labeled magnetically to function as an endogenous tracer [7]. Unlike other imaging techniques such as ultrasound, renal scintigraphy and angiography with contrast-enhanced CT or MRI, ASL enables both imaging and quantification of tissue perfusion. However, at the same time, this bears the lack of a gold standard to sufficiently validate this technique. It has been evaluated in several studies for both native and transplant kidneys in detecting perfusion changes in acute kidney injury, chronic kidney disease, renal artery stenosis as well as in acute allograft rejection and delayed graft function [8]. Concerning AVFs, to date, there has not been a documented case using ASL for the detection of renal AVFs, but several studies have demonstrated the beneficial use of ASL for the detection of AVFs in the brain [9–11] and perfusion measurement after embolization [12].

DWI is another emerging MRI technique for functional evaluation of the kidneys [10–13]. To visualize the random displacement of molecules by Brownian motion, strong gradients are applied with varying strength and duration encoding the sensitization to diffusion. IVIM is a concept that has been introduced to differentiate between diffusion and the perfusion fraction, also called pseudo-diffusion [14]. In several studies it has been adopted to evaluate renal allograft function for the early detection of graft rejection and functional deterioration [15, 16]. It has been demonstrated that the perfusion fraction derived from IVIM correlates closely with quantitative perfusion measurements assessed with ASL in renal allografts [17]. Similarly to ASL, there is no documented case applying IVIM for the detection of renal AVFs.

Despite their potential, ASL and IVIM have not yet been established in clinical routine for diagnostics of renal allografts. Recent technical developments improving image quality, signal-to-noise-ratio and reproducibility could bring these techniques a step closer to clinical translation. Imaging of renal allografts in particular could prove advantageous for different functional MRI techniques, since the most limiting factor, abdominal motion, is significantly reduced due the anatomical position of renal allografts in the pelvis. More studies with larger cohorts and longitudinal settings are needed to further evaluate the potential of functional MRI as a useful examination tool in case of renal allograft dysfunction and to monitor therapeutic interventions.

Unlike other imaging techniques such as contrast-enhanced ultrasounds, which has also been applied in several studies for the examination of transplant kidneys [18], these MRI techniques enable both objective imaging and quantification of the whole kidney perfusion. Though perfusion results are not specific for the detection of specific pathologies such as AVFs, they could support clinicians for a more focused application of diagnostic methods.

## Conclusion

Renal allograft dysfunction often represents complex problems for clinicians due to the need for diverse diagnostic methods and multiparametric approaches. In some cases, quantification of functional changes in renal allografts is needed beyond imaging. Multiparametric functional MRI with ASL and IVIM can detect and quantify alterations in parenchymal perfusion in kidney allografts with good sensitivity and therefore add valuable information to conventional imaging techniques regarding the cause of graft failure. For monitoring kidney allografts and therapeutic interventions, functional MRI may provide complementary information and improve allograft diagnostics, a suggestion which should be evaluated in further studies.

## Abbreviations

ASL: Arterial spin labeling
AVF: Arteriovenous fistula
CT: Computed tomography
DWI: Diffusion weighted imaging
PF: Perfusion fraction
IVIM: Intravoxel incoherent motion
MRI: Magnetic resonance imaging
RBF: Renal blood flow
